# Pegylated liposomal doxorubicin in combination with mitomycin C, infusional 5-fluorouracil and sodium folinic acid. A phase-I-study in patients with upper gastrointestinal cancer

**DOI:** 10.1038/sj.bjc.6601786

**Published:** 2004-04-13

**Authors:** R-D Hofheinz, A Willer, A Weisser, U Gnad, S Saussele, S Kreil, J T Hartmann, R Hehlmann, A Hochhaus

**Affiliations:** 1 Onkologisches Zentrum, III. Medizinische Klinik, Fakultät für Klinische Medizin Mannheim der Universität Heidelberg, Theodor-Kutzer-Ufer, D-68167 Mannheim, Germany; 2 II. Medizinische Klinik, Universitätsklinikum Tübingen, Germany

**Keywords:** gastric cancer, infusional 5-fluorouracil, mitomycin C, pancreatic cancer, pegylated liposomal doxorubicin, sodium folinic acid

## Abstract

Mitomycin C (MMC) in combination with infusional 5-fluorouracil (FU) plus folinic acid (FA) is an effective treatment for metastatic gastrointestinal cancer. Anthracyclines are commonly used in the treatment of upper gastrointestinal cancer. The aim of this study was to determine the maximum tolerated dose of liposomal, pegylated doxorubicin (Caelyx) in combination with infusional 5-FU/sodium FA and MMC. Escalating doses of Caelyx (15 – 25 – 30 – 35 mg m^−2^ corresponding to dose levels I–IV) were applied on days 1 and 29, given to fixed doses of 24-h 5-FU (2000 mg m^−2^) and sodium FA (500 mg m^−2^, mixed with 5-FU in one pump) weekly for 6 weeks, and MMC 7 mg m^−2^ on days 8 and 36. At least three patients were treated at each dose level. A total of 25 patients are evaluable. No dose-limiting toxicity (DLT) was observed on level I (*n*=3). On level II, DLT occurred in three out of five patients (mucositis and leucopenia). Owing to the early DLTs at this dose, we added a 20 mg m^−2^ Caelyx dose level (Ia). In total, 17 patients were treated at this dose level. Among these, only two patients experienced DLT in cycle one and 37 complete cycles have been administered in association with a low toxicity profile. The median dose intensity was 100% for each drug during the first course and no treatment delay exceeding 7 days was required. The recommended dose of 4-weekly Caelyx in combination with weekly 24-h 5-FU/sodium FA and 4-weekly MMC is 20 mg m^−2^. Preliminary antitumour activity has been observed in patients with pretreated pancreatic cancer and in untreated gastric cancer.

Liposomal encapsulation of anticancer drugs has been pursued intensively during the past number of years. This pharmaceutical strategy offers the theoretical advantage of increasing drug efficiency while decreasing toxicity. Pegylated liposomal doxorubicin (Caelyx) is a formulation of doxorubicin encapsulated in small stealth liposomes that confer a significantly longer half-life to the drug, and a particular tissue distribution with a high concentration of the active agent into the tumour. The dose-limiting toxicities (DLTs) when administered every 3 or 4 weeks at doses between 40 and 60 mg m^−2^ are hand–foot skin reaction and mucositis. In clinical trials, Caelyx has been associated with significantly less cardiac toxicity and myelotoxicity than free doxorubicin. Response to Caelyx has been demonstrated against a broad range of tumours (e.g. Kaposi's sarcoma, breast, ovarian, hepatocellular carcinoma, head and neck cancer). It could be shown that Caelyx can safely be combined with other cytotoxic drugs such as gemcitabine (Fracasso *et al*, 2002), vinorelbine (Gebbia *et al*, 2002), paclitaxel±cisplatin (Eng *et al*, 2001; Mavroudis *et al*, 2002) and docetaxel (Syrigos *et al*, 2002).

Only limited data is available on the efficacy of Caelyx in patients with gastric and pancreatic cancer. Six out of 16 evaluable patients with pancreatic cancer had stable disease upon treatment with single agent Caelyx (Halford *et al*, 2001). In gastric cancer, single agent Caelyx led to one partial remission and seven cases of stable disease in 24 evaluable patients (Thomas *et al*, 2001). Kagmakis *et al* (2000) combined 4-weekly Caelyx with bolus 5-fluorouracil (5-FU) and folinic acid (FA) for salvage treatment of gastrointestinal tumours. Remission or stable disease was observed in 60% of patients with pretreated gastric cancer.

Mitomycin (MMC), a quinone-containing antitumour antibiotic, has been used as a component of chemotherapy for gastrointestinal cancers for three decades, and there is evidence of *in vitro* synergy between MMC and 5-FU (Sartorelli and Boothe, 1965; Rusello *et al*, 1989). When combined with infusional 5-FU, objective remission rates up to 54% in gastric cancer patients have been observed (Hofheinz *et al*, 2002; Hartmann *et al*, 2003). In colorectal and pancreatic cancer, protracted infusional 5-FU plus MMC resulted in a superior response rate in comparison with 5-FU alone, and in an improved failure-free survival in colorectal cancer (Ross *et al*, 1997; Maisey *et al*, 2002).

This study aimed at determining the maximum tolerated dose and the DLTs of Caelyx in combination with MMC and weekly infusional 5-FU/FA in patients with upper gastrointestinal cancer. Sodium FA was used instead of calcium FA because it can be safely given simultaneously combined with 5-FU in a single infusion system (Hartung *et al*, 2001).

## PATIENTS AND METHODS

### Eligibility criteria

Patients with histologically confirmed gastrointestinal cancer refractory to at least one chemotherapy regimen were eligible. They should have been off previous anticancer therapy for at least 4 weeks. Patients with chemotherapy-naïve gastric cancer were eligible for inclusion at the recommended dose. Other eligibility criteria were an ECOG performance status ⩽2, age ⩽75 years, a life expectancy of at least 3 months and adequate bone marrow function (leucocyte count >3000 *μ*l^−1^, platelet count >100 000 *μ*l^−1^). Adequate renal (serum creatinine ⩽1.4 mg dl^−1^ or creatinine clearance >60 ml min^−1^) and hepatic function (bilirubin ⩽2 mg dl^−1^) were required. Left ventricular ejection fraction (LVEF), assessed by echocardiography, was at least 50% in eligible patients. Appropriate contraception was required in fertile patients. All patients provided written informed consent. The study protocol was reviewed and approved by the local institutional review board (IRB). Treatment was performed according to the Declaration of Helsinki.

### Staging procedures

Before admission to the study, all patients underwent a complete history, physical examination, ECG and chest X-rays. Cardiac ultrasonography was carried out to determine the LVEF. A full blood count with differential and serum chemistry was obtained within 14 days prior to the start of treatment.

During the study period, weekly blood count monitoring was performed and serum chemistry was repeated fortnightly. LVEF was assessed before the initiation of a new treatment cycle.

Toxicities were recorded weekly and graded according to the Common Toxicity Criteria (CTC) of the National Cancer Institute (version 2.0).

Although patients were not required to have bidimensionally measurable disease to enter the study, computed tomography (CT) scans of the tumour-bearing region were performed within 4 weeks prior to the start of study treatment. Indicator lesions were assessed every 8 weeks according to WHO standard criteria.

### Treatment schedule, dose escalation and study design

Treatment was given weekly for a total of 6 weeks followed by a 2-week rest period. Therefore, one course equalled 57 days. The chemotherapy regimen consisted of a weekly 24-h continuous infusion of 5-FU 2000 mg m^−2^ mixed with sodium FA 500 mg m^−2^ in a portable pump. A central venous port system was implanted in all patients. Bolus MMC was administered at a dose of 7 mg m^−2^ on days 8 and 36. Dexamethasone 8 mg was added intravenously to MMC to prevent pulmonary toxicity. Caelyx was planned as a 1-h infusion on days 1 and 29 at a dose of 15 mg m^−2^ (dose level I), 25 mg m^−2^ (dose level II), 30 mg m^−2^ (dose level III) and 35 mg m^−2^ (dose level IV). Owing to DLTs on dose level II in three out of five patients, another dose level (Ia; Caelyx 20 mg m^−2^) was added and approved by the IRB. Dexamethasone 8 mg and clemastine 0.5 mg were added routinely to Caelyx after the observation of allergic reactions in two patients during the first Caelyx administration. All patients received a standard i.v. antiemetic prophylaxis (ondansetron or tropisetron) to avoid a bias on gastrointestinal toxicity.

Dose-limiting toxicity was defined during the first cycle of chemotherapy (i.e. within the first 57 days), by the occurrence of one of the following toxicities: grade 4 leucopenia/neutropenia or thrombocytopenia, symptomatic thrombocytopenia (haemorrhage), grade 3 or 4 febrile neutropenia, any ⩾grade 3 nonhaematological toxicity except nausea/vomiting. At least three patients were enrolled per dose level, and the number was increased to six if DLT occurred in at least one of the first three. Escalation continued only if DLT was limited to one of six patients. Escalation halted if DLT occurred in two or more patients. Maximum tolerated dose (MTD) was defined as the highest dose at which fewer than two of three or six patients experienced DLTs during the first course of chemotherapy. Individual dose escalation was not allowed.

To expand the toxicity data and to assure the safety of this combination, the recruitment of further patients at the MTD was provided for in the study protocol. It was planned to also enrol chemotherapy-naïve patients with gastric cancer, because even infusional 5-FU/FA in combination with MMC represents an effective treatment for these patients, thus making undertreatment by our investigational regimen unlikely.

Dose intensity at the MTD was determined by dividing the actually delivered cumulative doses of each drug by the scheduled cumulative doses.

The overall survival calculation used death due to any reason as the end point (Kaplan and Maier, 1958).

## RESULTS

### Patient characteristics

A total of 27 patients were enrolled in the study between July 2001 and November 2002. A total of 25 patients were evaluable for toxicity according to the protocol. The remaining two patients were withdrawn from the study for the following reasons: One patient discontinued therapy after the third administration of 5-FU due to the development of a bilioma that required surgical intervention. Another patient experienced an anaphylactic reaction with bronchospasm, dyspnoea and severe back pain upon the first administration of Caelyx. In view of this event, therapy was continued without Caelyx.

The characteristics of all enrolled patients are described in Table 1

**Table 1:** Patient demographics

**Variable**	**No. of patients (%)**
Enrolled	27 (100)
Assessable	25 (93)
Median age	63 (range, 38–74)
	
*Gender*	
Male	16 (59)
Female	11 (41)
	
*Performance status (ECOG)*	
Median	1
0	1 (4)
1	17 (63)
2	9 (33)
	
*Tumor origin*	
Pancreatic cancer	12 (44)
Gastric cancer	11 (41)
Cholangiocarcinoma	3 (11)
Cancer of unknown primary (adenocarcinoma)	1 (4)
	
*Prior chemotherapy*	
None (gastric cancer)	10 (37)
One line	13 (48)
Two lines	4 (15)
	
Gemcitabine	15 (56)
5-fluorouracil-based (i.v. or oral)	8 (30)
Others (irinotecan, oxaliplatin)	2 (7)
	
*Tumor localisation*	
Primary	19 (70)
Liver	17 (63)
Lymph nodes	6 (22)
Peritoneal carcinomatosis	10 (37)
Other	2 (7)

i.v.=intravenous.

. A total of 12 patients with pancreatic cancer had received at least one previous chemotherapy regimen (gemcitabine±5-FU/FA). Tumour at the primary site, liver metastases as well as peritoneal carcinomatosis were the most common tumour sites.

After the determination of the MTD, chemotherapy-naïve patients with metastatic gastric cancer were treated at the recommended dose. These patients generally had unresectable cancer with peritoneal spread of their disease.

### Determination of the MTD and dose intensity

Three patients were treated on dose level I (Caelyx 15 mg m^−2^). They received a total of eight cycles of chemotherapy (1, 3 and 4 cycles, respectively). Dose-limiting toxicities were neither observed in cycle 1 nor in any subsequent cycles (Tables 2

**Table 2:** Haematological toxicity (according to Common Toxicity Criteria) per dose level in cycle one and in subsequent cycles (worst per patient)

	**Toxicity (no. of patients)**												
**Dose level**	**No. of patients**	**Anaemia**				**Leucopenia**				**Thrombocytopenia**			
*Cycle 1*													
		**1**	**2**	**3**	**4**	**1**	**2**	**3**	**4**	**1**	**2**	**3**	**4**
I	3	1	1	1	—	1	—	—	—	—	—	—	—
Ia	17	12	4	—	—	1	3	1	1	6	—	—	—
II	5	2	3	—	—	—	—	2	1	—	—	3	—
													
*Cycles* >*1*													
		**1**	**2**	**3**	**4**	**1**	**2**	**3**	**4**	**1**	**2**	**3**	**4**
I	2	1	1	—	—	—	—	—	—	1	—	—	—
Ia	12	7	5	—	—	4	1	1	—	7	—	1	—
II	2	1	1	—	—	—	—	—	—	—	1	—	—

and 3

**Table 3:** Nonhaematological toxicity (according to Common Toxicity Criteria) per dose level in cycle 1 and in subsequent cycles (worst per patient)

	**Toxicity (no. of patients)**														
**Dose levels**	**No. of patients**	**Nausea/emesis**			**Stomatitis/mucositis**			**Diarrhoea**			**Alopecia**		**Hand–foot skin reaction**		
*Cycle 1*															
		**1/2**	**3**	**4**	**1/2**	**3**	**4**	**1/2**	**3**	**4**	**1**	**2**	**1**	**2**	**3**
I	3	2	—	—	—	—	—	1	—	—	—	—	—	—	—
Ia	17	8	5	—	7	1	1	4	2	—	4	1	4	—	—
II	5	1	—	—	—	3	—	—	1	—	1	1	—	—	1
															
*Cycles* >*1*															
		**1/2**	**3**	**4**	**1/2**	**3**	**4**	**1/2**	**3**	**4**	**1**	**2**	**1**	**2**	**3**
I	2	2	—	—	1	—	—	1	—	—	—	—	—	—	—
Ia	12	9	—	—	5	—	—	6	1	—	3	1	2	2	—
II	2	1	—	—	—	—	—	1	1	—	—	1	—	1	—

). However, three of five patients on dose level II (Caelyx 25 mg m^−2^) experienced DLT between treatment days 14 and 16. One patient experienced grade 4 leucopenia and two patients grade 3. Grade 3 thrombocytopenia was noticed in three patients. Grade 3 mucositis/stomatitis – with additional diarrhoea in one patient – was dose limiting in these three patients as well.

Owing to the unexpected early occurrence of DLT at dose level II and the rather big gap in Caelyx dosage between levels I and II, we decided to amend the study protocol and include a dose level Ia (Caelyx 20 mg m^−2^). The amendment was approved by the IRB.

One of the first six patients enrolled at dose level Ia experienced DLT (leucopenia grade 4, mucositis grade 4 and diarrhoea grade 3). Therefore, dose level Ia was determined as the MTD. A total of 17 patients were treated at this dose level, including 10 patients with chemotherapy-naïve gastric cancer. Only two of these patients – including the patient mentioned above – developed DLT during cycle one. In all, 37 complete cycles of chemotherapy were given to these 17 patients. A total of 16 patients completed cycle 1. A total of 12 patients have so far received at least two and six patients at least three complete cycles of chemotherapy.

**Figure 1:**
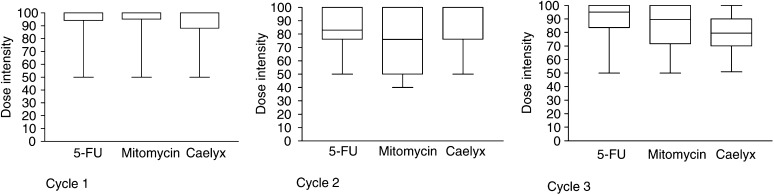
Dose intensity (in percentage) of each compound in patients treated at the recommended dose during cycles one (*n*=17), two (*n*=12) and three (*n*=6), respectively

Dose intensity was calculated for all patients treated at dose level Ia (Figure 1

). A median dose intensity of 100% was reached in cycle one for each drug (mean dose intensity: 5-FU/FA 94%, MMC 94%, Caelyx 88%). Treatment delays were required only in five patients during cycle one, which did not exceed 7 days in each case. The median dose intensities for each drug in cycle two (*n*=12 patients) and three (*n*=6 patients) remained high.

### Toxicity

Haematological and nonhaematological toxicities are summarised separately for the first and subsequent cycles in Tables 2 and 3. No DLT occurred during cycle 1 or subsequent cycles in three patients treated on dose level I, but DLT was observed in three out of five patients on dose level II. Each of these patients experienced leucopenia ⩾grade 3, thrombocytopenia grade 3 and gastrointestinal toxicities (mucositis grade 3, *n*=3 and diarrhoea grade 3, *n*=1). After reducing the Caelyx dose to 20 mg m^−2^ (level Ia), DLTs were observed in only two out of 17 patients (12%) in cycle one. During 37 treatment cycles administered at the recommended dose level to 17 patients, only three patients (18%) showed DLTs (cycle one *n*=2, cycle three *n*=1) (Table 4

**Table 4:** Toxicity (worst per patient) for all patients treated at dose level Ia (Caelyx 20 mg^−2^) according to Common Toxicity Criteria (*n*=17 patients; *n*=37 cycles)

**Toxicity**	**Grade I/II (%)**	**Grade III (%)**	**Grade IV (%)**
Anaemia	17 (100)	0	0
Leucopenia	6 (35)	1 (6)	1 (6)
Thrombocytopenia	9 (53)	1 (6)	0
Nausea/emesis	8 (47)	5 (29)	0
Stomatitis/mucositis	7 (41)	1 (6)	1 (6)
Diarrhoea	9 (53)	3 (18)	0
Alopecia	6 (35)	—	—
Hand–foot skin reaction	4 (24)	0	—

). No thrombocytopenia grade 3 or 4 was observed during cycle one at dose level Ia and leucopenia ⩾grade 3 was documented in only two patients. Caelyx 20 mg m^−2^ in combination with infusional 5-FU/FA and MMC proved to be safe and well tolerated even in subsequent courses of chemotherapy. Interestingly, hand–foot skin reaction – regarded as a manifestation of overlapping toxicity of infusional 5-FU and Caelyx – was observed in only four patients treated at the recommended dose level (24%), although the investigators were vigilant to the possibility of this adverse event and no prophylactic measures (e.g. vitamin B6) were in place. In our series, only one patient required a dose reduction because of hand–foot skin reaction.

No renal toxicity and no evidence of MMC-induced haemolytic–uremic syndrome was seen.

### Antitumour activity

A total of 17 patients had bidimensionally measurable tumour masses. Two objective responses (pancreatic cancer and chemotherapy-naïve gastric cancer) and two minor remissions (pancreatic cancers) were observed. Of the 11 patients with advanced gastric cancer, only three had measurable disease (partial remission (PR), *n*=1 and no change (NC), *n*=2). Median survival in these patients has not yet been reached after a median follow-up of 9 months, despite the fact that the vast majority of patients had tumours with unfavourable prognostic characteristics (irresectable primary and peritoneal carcinomatosis). The group of patients with pancreatic cancer failing at least one previous line of chemotherapy had a median survival of 6.5 months calculated from the start of study treatment (i.e. second- or third-line chemotherapy).

## DISCUSSION

Combinations of infusional 5-FU/FA with MMC are commonly used in gastrointestinal cancer (Hartmann *et al*, 2003). In gastric cancer, MMC (10 mg m^−2^) added every third week to weekly infusional 5-FU/FA yielded a response rate of 54%, and a median overall survival of 10.2 months (Hofheinz *et al*, 2002). Ross *et al* (2002) reported the results of a randomised study comparing ECF (3-weekly epirubicin and cisplatin in combination with protracted venous infusional (PVI) 5-FU 200 mg m^−2^ daily) to MCF (MMC 7 mg m^−2^ every 6 weeks and 3-weekly cisplatin with PVI-5-FU 300 mg m^−2^ daily). The MCF and ECF regimen resulted in equivalent response rates and survival, thus confirming the efficacy of combined infusional 5-FU and MMC in gastric cancer.

For patients with metastatic colorectal cancer, several studies on combination therapy of MMC and infusional 5-FU in first- or second-line protocols have been published (e.g. Ross *et al*, 1997; Chester *et al*, 2000; Grumett *et al*, 2001). Administered as salvage treatment, the addition of MMC to 5-FU-containing regimens may overcome resistance to 5-FU.

In both, colorectal and pancreatic cancer, protracted infusional 5-FU plus MMC resulted in a superior response rate in comparison with 5-FU alone, but this did not translate into a survival advantage (Ross *et al*, 1997; Maisey *et al*, 2002).

Anthracyclines are used in combination regimens in gastric cancer (e.g. Ross *et al*, 2002) or pancreatic cancer (e.g. Neri *et al*, 2002). They formed part of the FAM (5-FU, doxorubicin, MMC) and FAMtx (5-FU, doxorubicin, methotrexate) regimens, respectively. Either of these combinations was formerly considered standard treatment in gastric cancer. The FAM has been shown to be superior to best supportive care in pancreatic carcinoma (Palmer *et al*, 1994), although trials have failed to clearly demonstrate an advantage over single agent 5-FU (Cullinan *et al*, 1985).

No combination trial with Caelyx and infusional 5-FU±MMC has been published to date. Based on the outcomes of previous studies, it seemed rationale to test the combination of Caelyx with infusional 5-FU, FA and MMC with the aim of identifying a regimen to deliver 5-FU, MMC and doxorubicin in their presumably most effective manner: infusional 5-FU modulated by sodium FA (mixed in one pump), dose-dense MMC and liposomal pegylated doxorubicin. The phase I study presented here was designed to evaluate the safety and tolerability of escalating doses of Caelyx in combination with weekly infusional 5-FU/sodium FA and 4-weekly bolus MMC. 5-Fluorouracil and MMC were planned for use at 75% of the doses administered in our former studies (Hofheinz *et al*, 2002; Hartmann *et al*, 2003) and the Caelyx dose was escalated.

Four-weekly Caelyx 20 mg m^−2^ can safely be combined with weekly infusional 5-FU/sodium FA and 4-weekly MMC. Mucositis and leucopenia were the dose-limiting side effects. A total of 37 cycles have been applied to 17 patients at the recommended dose level. Dose-limiting toxicity was observed in only three of these patients during the first cycle or subsequent cycles. A median dose intensity of 100% was reached for each drug during cycle one (57 days). Therapy delays were fairly uncommon and did not exceed 1 week. Despite the limited number of patients treated so far with this combination therapy, we conclude that a high dose intensity can be achieved in a considerable percentage of patients for periods of up to 6 months. Neither renal toxicity, haemolytic uremic syndrome, nor pulmonary toxicity has been observed. Hand–foot skin reaction, a painful desquamating dermatitis of the hands and feet, is seen in association with infusional 5-FU as well as with Caelyx. It has only been observed in 24% of the patients treated at the recommended dose level in our study. Apparently, the combination of both drugs at the investigated doses does not confer a higher incidence of hand–foot skin reaction than either drug alone. Therefore, it might be speculated that different pathogenetic mechanisms are involved.

Despite administering an obviously well-tolerated regimen, we noticed a median survival of 6.5 months in patients with pretreated pancreatic cancer. This might be biased due to patient selection. Tumour shrinkage was seen in three of these patients. A total of 10 patients with metastatic gastric cancer, mostly with peritoneal spread not amenable to objective tumour assessment, have been treated for first line. One PR and two NC lasting for 8 months were observed in three patients with measurable disease. The median survival has not yet been reached.

In conclusion, the established application schedule for the combination of Caelyx, MMC and weekly 24-h infusional 5-FU/sodium FA is well tolerated and appears to be active in patients with upper gastrointestinal cancer. High dose intensity was achieved in a substantial percentage of patients for several months. Our current efforts focus on phase II studies in gastric and pretreated pancreatic cancer.
